# Bridging internalized HIV stigma and depressive symptoms among people living with HIV in China during the COVID-19 pandemic: a network analysis

**DOI:** 10.3389/fpubh.2023.1306414

**Published:** 2024-01-05

**Authors:** Guangzhe F. Yuan, Shan Qiao, Xiaoming Li

**Affiliations:** ^1^School of Education Science, Leshan Normal University, Leshan, Sichuan, China; ^2^Arnold School of Public Health, University of South Carolina, Columbia, SC, United States

**Keywords:** internalized HIV stigma, depressive symptoms, PLWH, COVID-19, network analysis

## Abstract

Previous research has documented that HIV-related stigma may be a significant trigger of mental health problems among people living with HIV (PLWH). However, less is known about how internalized HIV stigma is linked to depressive symptoms among PLWH during the COVID-19 pandemic. The current study sought to explore the network structure of internalized HIV stigma and depressive symptoms, along with bridge nodes, to elucidate how they co-exist. Participants were 1,197 Chinese PLWH (64.3% male, *M*_age_ = 41.52, SD = 9.20) who completed the measurements of internalized HIV stigma and depressive symptoms during the early phase of the COVID-19 outbreak (May 2020). Results revealed that “ashamed of having HIV” was identified as the most central nodes in the internalized HIV stigma network, whereas “mind wandered during tasks” ranked highest on centrality in the depressive symptoms network. Two bridge connections were exhibited within the combined internalized HIV stigma and depressive symptoms network model: “inferiority due to HIV” and “gloomy feelings” from internalized HIV stigma and depressive symptoms communities, respectively. This study is one of the first to examine the co-occurrence of internalized HIV stigma and depressive symptoms in the context of the COVID-19 pandemic using a network approach. These findings have potential clinical implications for mitigating depressive symptoms in populations facing socioeconomic disadvantage and vulnerability.

## 1 Introduction

The COVID-19 pandemic has underscored the intersection of public health crises and mental health challenges, particularly among disadvantaged and vulnerable populations such as people living with HIV (PLWH) (1). The added strain of the pandemic has exacerbated mental health problems, particularly depressive symptoms, among this group (2). Depressive symptoms, including persistent sadness, lack of interest in daily activities, cognitive difficulties, and physical malaise, are prevalent in PLWH due to the chronic nature of their condition (3–5). These symptoms may be exacerbated by the stressors and uncertainties of the COVID-19 pandemic, such as increased social isolation, fear of increased vulnerability to the virus, and disruptions in health care services (6, 7). A recent systematic review and meta-analysis study found that the pooled prevalence rate of (moderate-to-severe) depressive symptoms among PLWH during the COVID-19 was 16.9% [95% confidence interval (CI): 3.8–30.0%] (6). Winters and colleagues compared the prevalence of depression among PLWH in Shinyanga region, Tanzania before and during COVID-19 and found substantially higher prevalence of depression (prevalence differences: 38; CI: 34, 42) (8). Given the existing health complications and increased risk of mental health issues, understanding the interplay between predictive factors and depressive symptoms among PLWH during COVID-19 is of paramount importance to enable more tailored and effective interventions for this population.

Internalized HIV stigma has been found to be one of the most significant risk factors of depressive symptoms among PLWH (9, 10). This stigma involves the incorporation of negative societal attitudes about HIV, resulting in self-disparagement, feelings of rejection and guilt (11). The harmful self-perceptions that individuals hold may serve as a direct antecedent to depressive symptoms. This occurs as individuals cultivate a negative self-concept and engage in harmful self-evaluations (12). In support of Meyer’s minority stress theory, which emphasizes the consequential role of stigma in generating psychological distress, individuals who belong to disadvantaged minority groups, such as PLWH, exhibit greater susceptibility to both external and internal stressors (13). These stressors are inextricably intertwined with instances of stigma and discrimination, making PLWH more vulnerable to depressive symptoms than their non-HIV peers (13).

Simultaneously, the COVID-19 pandemic may exacerbate internalized HIV stigma in addition to depressive symptoms (14). The additional stigma associated with the pandemic may intersect with and reinforce existing HIV-related stigma, exacerbating depressive symptoms (2). Because of their interrelated nature, understanding the complex relationship between internalized HIV stigma and depressive symptoms in PLWH during the COVID-19 pandemic is critical. Internalized stigma may exacerbate depressive symptoms through feelings of isolation, self-blame, and anticipated rejection. Conversely, the presence of depressive symptoms may increase the experience of internalized stigma, as individuals may further blame themselves for their condition (15). Additionally, existing evidence suggests that several underlying problems experienced by PLWH during COVID-19 may exacerbate the incidence of internalized stigma and depressive symptoms. For example, a scoping review of 45 articles found that several factors were related to increased psychological distress during the pandemic, including substance use, social support, financial hardship, antiretroviral adherence, and economic vulnerability (16). A recent qualitative study revealed that medical mistrust among PLWH might be associated with HIV stigma during the COVID-19 pandemic (17). It is therefore important for HIV researchers to explore the potential relationship between internalized HIV stigma and depressive symptoms during the pandemic in this population.

Although numerous empirical studies have documented the strong association between internalized HIV stigma and depressive symptoms, there is still a research gap regarding the network analysis model between internalized HIV stigma and depressive symptoms among PLWH during the COVID-19 pandemic. In this study, we attempted to use a novel analytical method—psychometric network analysis—to explore the relationship between these two variables in the context of the COVID-19 pandemic.

In contrast to traditional approaches that treat internalized HIV stigma and depressive symptoms as latent variables that are independent of each other, network analysis offers an alternative methodology capable of visualizing and quantifying the complexity inherent in such a system of interrelated variables (18–21). It provides a detailed understanding of these complex interactions and sheds light on the interplay between internalized HIV stigma and depressive symptoms within a larger context of psychosocial factors (22, 23). Using network analysis, elements of internalized HIV stigma and depressive symptoms are conceptualized as “nodes,” while the relationships between these elements are referred to as “edges” (24). When these nodes coexist within a population, they are considered to be directly connected. These critical links that serve as conduits between internalized HIV stigma and depressive symptoms are referred to as “bridges” in the network model (25).

Applied to the context of PLWH, this methodology can highlight specific attributes of internalized HIV stigma or specific depressive symptoms that exert a significant influence within the network. This identification is important for intervention development, as strategies can be tailored to address these key nodes, potentially leading to a more profound system-wide impact. In addition, network analysis helps to demystify the potential pathways linking internalized HIV stigma and depressive symptoms. Understanding these pathways is useful in creating predictive models, allowing clinicians and researchers to anticipate the progression of these symptoms based on network models (26). Such insights could provide timely indications for intervention, potentially averting further psychological distress. Therefore, network analysis can serve as a valuable tool for understanding and addressing the intertwined relationship between internalized HIV stigma and depressive symptoms among PLWH. By providing a nuanced and targeted understanding of this relationship, it can inform more effective strategies to alleviate psychological distress during challenging public health crisis in the future.

### 1.1 The current study

In the present study, we utilized network analysis to construct three different models: a network for internalized HIV stigma, a network for depressive symptoms, and a combined network model that includes both internalized HIV stigma and depressive symptoms. The objective was to identify critical “bridging nodes” that is the fundamental elements providing connections between internalized HIV stigma and depressive symptoms. Because network analysis is fundamentally a method for revealing key relationships within and across groups of nodes, we did not hypothesize about the particular nodes or edges that might emerge as central in these network models.

## 2 Materials and methods

Data were drawn from a large cohort research project that attempted to explore the behavioral and mental health problems of PLWH in Guangxi, China (15, 27). The data were collected from May 2020 to October 2020. In collaboration with the Guangxi Center for Disease Control and Prevention (CDC), a strategic selection of study sites was made to conduct the research. We identified six hospitals and clinics in five cities based on their high numbers of HIV patients under care. This selection allowed us to focus on the sites with the largest HIV patient populations, thereby increasing the scope and relevance of our study. All participants were recruited from these local HIV clinics according to their medical records. This study was designed with precise inclusion criteria for participants: individuals who are aged between 18 and 60, diagnosed with HIV/AIDS, and have no plans to relocate from Guangxi for subsequent follow-up investigations lasting more than 12 months. Excluded from participation were those with language, mental, or physical impairments that might affect their ability to respond to assessment questions; those currently detained or institutionalized for drug use or involvement in commercial sex; or those intending to leave the province within the next year. In total, the study included 1,197 PLWH who successfully completed the survey. Sample demographics are shown in Table 1.

**Table 1:** Sample characteristics (*n* = 1,197).

Variables	*M* (SD) or *n* (%)
Age	41.52 (9.20)
**Sex**	
Female	427 (35.7%)
Male	770 (64.3%)
**Ethnic group**	
Han	779 (65.1%)
Other	418 (34.9%)
**Residence**	
Urban	535 (44.7%)
County	111 (9.3%)
Township	120 (10.0%)
Rural	428 (35.8%)
**Marital status**	
Never married	323 (27.0%)
Unmarried—living with partner	25 (2.1%)
Married	641 (53.6%)
Married—separated	36 (3.0%)
Divorced	93 (7.8%)
Widowed	67 (5.6%)
**Education**	
Primary school	217 (18.1%)
Middle school	467 (39.0%)
High school	238 (19.9%)
College	228 (19.0%)
Other	47 (3.9%)
**Employment status**	
Unemployed	221 (18.5%)
Part-time	235 (19.6%)
Full-time	732 (61.2%)
**Monthly household income (CNY)**	
0–999	122 (10.2%)
1,000–1,999	247 (20.6%)
2,000–2,999	350 (29.2%)
3,000–3,999	234 (19.5%)
4,000–4,999	93 (7.8%)
5,000 or more	148 (12.4%)

The number of participants for some variables does not add up to the total sample size due to missing data.

Ethical approval to conduct this study was granted by the Institutional Review Boards of University of South Carolina. The study procedures were fully disclosed to all participants, who were informed of the purpose of the study, the confidentiality of their responses, and their rights as participants. This information included their freedom to withdraw from the study at any time. After providing informed consent, participants were asked to complete a questionnaire administered by an interviewer. The interview-style questionnaires were administered by nurses trained by experts with more than 10 years of research experience with PLWH. In recognition of their contribution to the study, participants were offered an incentive of US$5.00 (equivalent to approximately 35 RMB, the Chinese currency).

### 2.1 Measures

#### 2.1.1 Depressive symptoms

Depressive symptoms were assessed by a Chinese version of the Center of Epidemiological Studies Depression Scale (CESD-10) (28). The questionnaire contains 10 items, each rated on a four-point scale from 1 (rarely or none of the time) to 4 (all the time). Sample items include “I had trouble keeping my mind on what I was doing” and “My sleep was restless.” This questionnaire has shown good reliability and construct validity in Chinese samples (29, 30). A higher total score indicates a higher level of depressive symptoms. Cronbach’s alpha reliability for the CESD-10 in this study was 0.81.

#### 2.1.2 Internalized HIV stigma

The assessment of internalized HIV stigma was conducted using an adapted Chinese version of eight items from the Negative Self Image Scale (31). Previous research with Chinese PLWH has demonstrated satisfactory validity and reliability for the Chinese version of this questionnaire (32, 33). Examples of the items include, “Having HIV makes me feel like I’m a bad person” and “I feel guilty because I have HIV.” This instruction consists of eight items, each rated on a four-point scale from 1 (strongly disagree) to 4 (strongly agree). A higher total score indicates a higher level of internalized HIV stigma. In the current study, the scale demonstrated excellent internal consistency, Cronbach’s alpha reliability was 0.94.

To ensure relevance to the COVID-19 context, we supplemented these with some COVID-19-specific instructions. This approach allowed us to capture the nuanced ways in which the pandemic has affected depressive symptoms and internalized stigma, despite the generic nature of these instruments.

### 2.2 Data analysis

Data management, univariate statistics, and network analyses were performed using SPSS 23.0 and R software version 4.2.2. Analyses of the distributions of internalized HIV stigma and depressive symptoms indicated that they did not deviate significantly from normality. Specifically, assessments of skewness and kurtosis-measures were used to describe the shape and distribution of the data-indicated nominal deviations. The absolute values for skewness ranged from 0.01 to 2.19, while those for kurtosis ranged from 0.05 to 5.66. These results suggest that the data for internalized HIV stigma and depressive symptoms were reasonably well distributed, allowing for the subsequent use of parametric statistical methods (34).

We implemented the graphical Least Absolute Shrinkage and Selection Operator (LASSO) method to establish regularized partial correlation networks (24). These networks were then visualized using the *qgraph* package (35). We assessed the centrality of each node within these networks by calculating the Expected Influence (EI), an effective centrality index. This step allowed us to identify the most influential nodes within each network model (36). In our approach, we created separate regularized partial correlation networks for internalized HIV stigma and depressive symptoms. We then merged these into a combined network model to examine their potential coexistence (20, 37). To understand the interplay between internalized HIV stigma and depressive symptoms within the combined network model, we used the *networktools* package. This tool facilitated the calculation of the Bridge Expected Influence (BEI), which allowed us to identify which nodes served as “bridges” between these two phenomena (38). Nodes with the highest BEI values were designated as bridge nodes in this model (38). To ensure the reliability of our network models, we used the *bootnet* package. We performed 1,000 case-dropping bootstraps to calculate the correlation stability coefficient (CS-coefficient), which measures the stability of each network model (39). Following previous researchers’ suggestions (24, 40), we used a CS-coefficient cutoff of 0.25 and set the default value for the bootstrapping procedure at *r* = 0.7.

## 3 Results

Descriptive statistics for this study, including means, standard deviations, and intercorrelations of the variables of interest, are detailed in Table 2. In addition, Table 3 describes the names of the nodes used in the network analysis for internalized HIV stigma and depressive symptoms, and presents univariate statistics for each node.

**Table 2:** Means, standard deviations, and correlations among study variables.

	*M* ± SD	1	2
1. Internalized HIV stigma	16.31 ± 5.46	–	0.25^***^
2. Depressive symptoms	16.73 ± 4.17	0.25^***^	–

Left/bottom triangle is the Pearson’s correlations of all study variables, right/top triangle is the partial correlations of all the variables adjusted by age, sex, and monthly household income. ^***^p < 0.001.

**Table 3:** Descriptions and univariate statistics for network nodes.

Node	Item content	*M*	SD
IHS1	HIV self-perceived badness	2.06	0.81
IHS2	Ashamed of having HIV	2.10	0.83
IHS3	Unclean feeling due to HIV	2.03	0.81
IHS4	Inferiority due to HIV	2.13	0.84
IHS5	Self-degradation due to HIV	1.92	0.76
IHS6	HIV-associated guilt	1.95	0.80
IHS7	HIV-related disgust	1.89	0.78
IHS8	HIV stigma worsens self-perception	2.23	0.88
DEP1	Unusual bothersomeness	1.27	0.53
DEP2	Mind wandered during tasks	1.37	0.62
DEP3	Gloomy feelings	1.42	0.67
DEP4	Increased effort in tasks	1.43	0.69
DEP5	Negative future optimism	2.48	1.10
DEP6	Experiencing fear	1.40	0.63
DEP7	Restless sleep	1.57	0.77
DEP8	Unhappiness	2.59	1.00
DEP9	Loneliness	1.60	0.75
DEP10	Lack of motivation	1.59	0.75

### 3.1 Internalized HIV stigma network model

**Figure 1:**
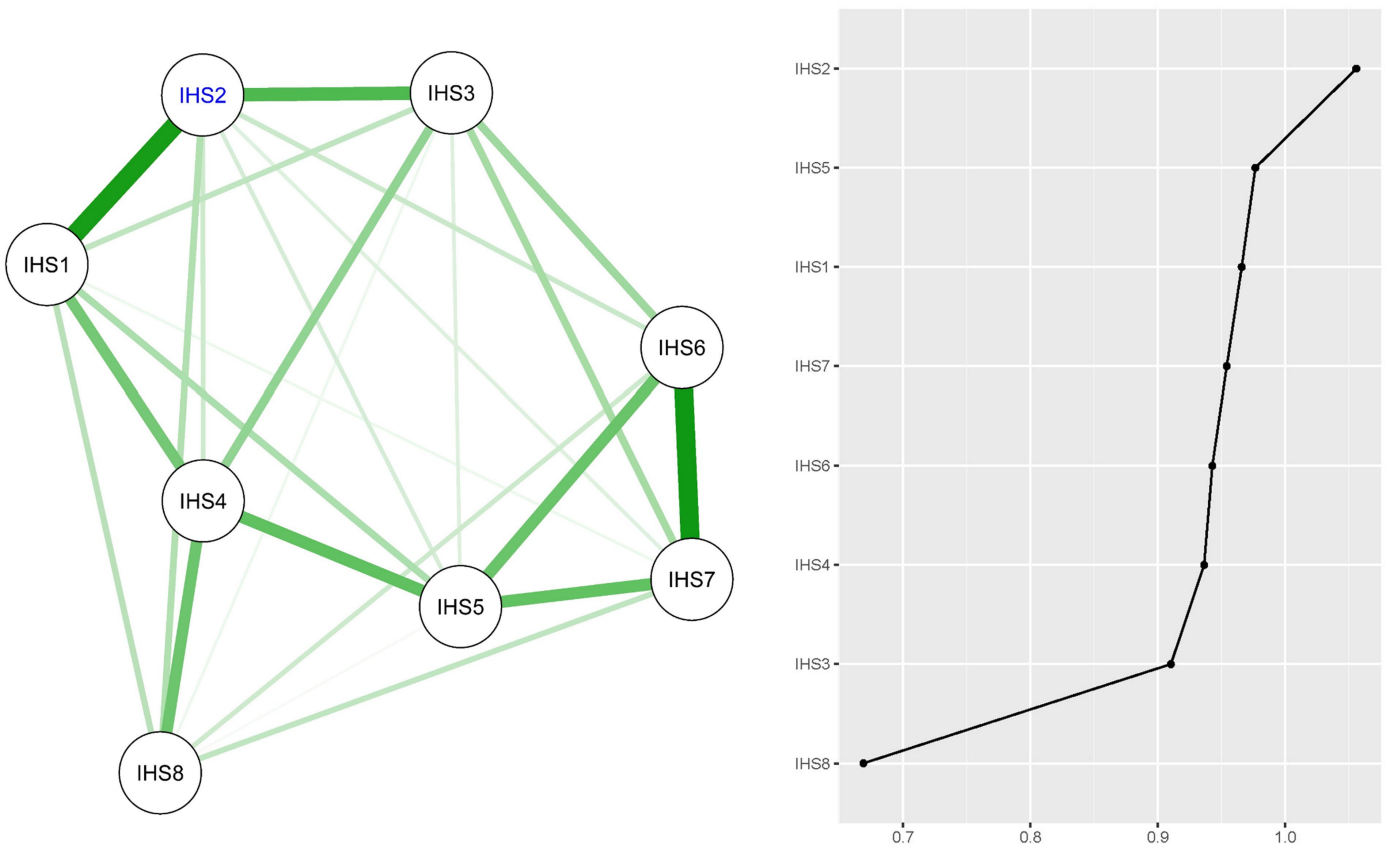
Regularized partial correlation network model for internalized HIV stigma (IHS) and expected influence (EI) values for each node. Node with largest EI value is represented by blue text (IHS2).

Figure 1 provides a visualization of the network structure of internalized HIV stigma, including the EI value for each node. The stability of the internalized HIV stigma network model is confirmed by acceptable CS-coefficients for edge weights [CS (cor = 0.7) = 0.75] and EI values [CS (cor = 0.7) = 0.74], according to the stability criteria (24). Within this model, the symptom “Ashamed of having HIV” (IHS2) emerged as the most influential node, followed by “Self-degradation due to HIV” (IHS5). The symptom with the least centrality was “HIV stigma worsens self-perception” (IHS8). The strongest edges were between “HIV self-perceived badness” (IHS1) and “Ashamed of having HIV” (IHS2), and between “HIV-associated guilt” (IHS6) and “HIV-related disgust” (IHS7). Supplementary Figure S1 shows the standardized estimates of node strength, betweenness, closeness, and expected influence, while Supplementary Figure S2 shows the bootstrapped confidence intervals (CIs) for the edge weights.

### 3.2 Depressive symptoms network model

**Figure 2:**
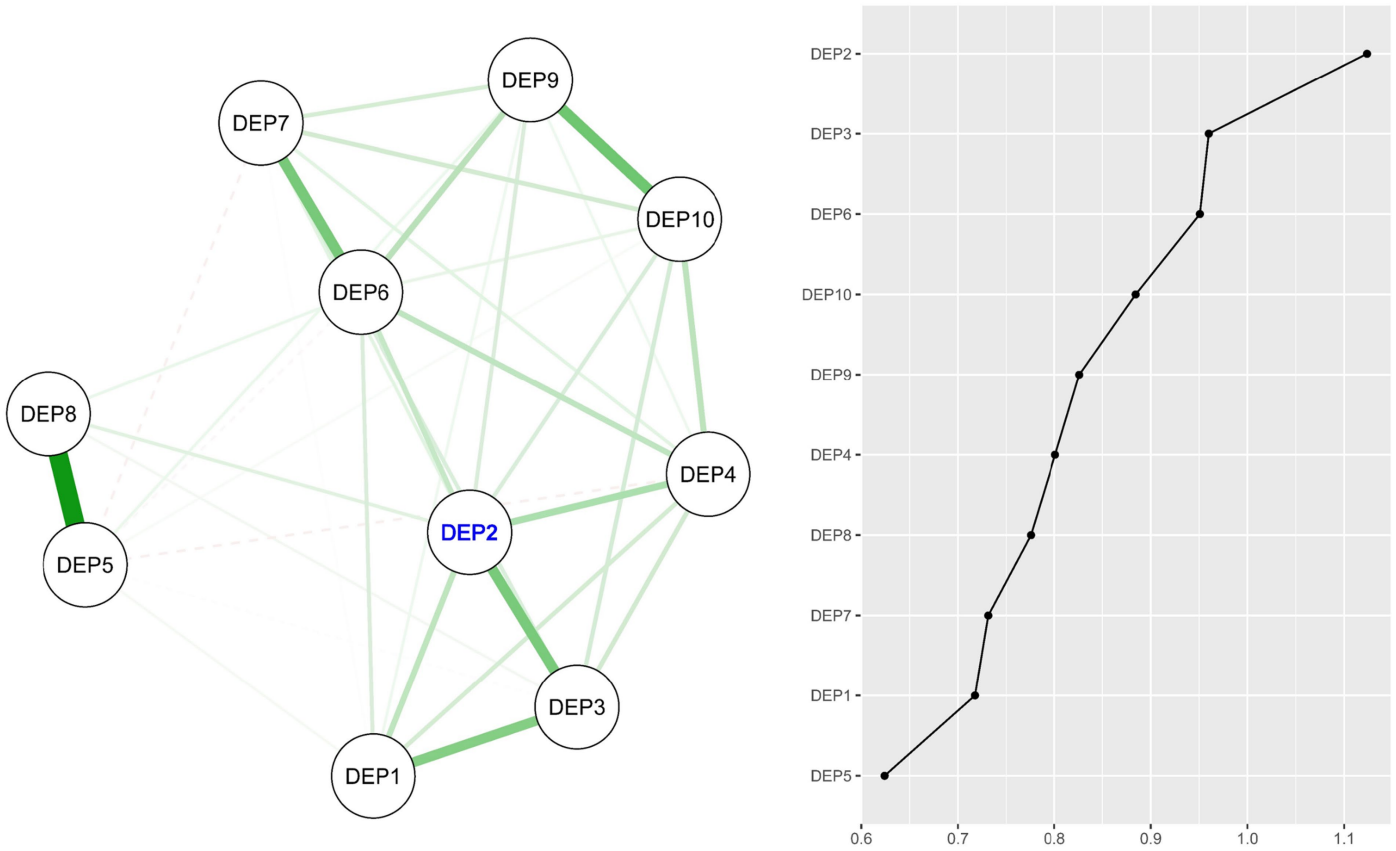
Regularized partial correlation network model for depressive symptoms (DEP) and expected influence (EI) values for each node. Node with largest EI value is represented by blue text (DEP2).

The network model of depressive symptoms is shown in Figure 2, with edge weights [CS (cor = 0.7) = 0.75] and EI values [CS (cor = 0.7) = 0.75] confirming a stable network model of depressive symptoms. The symptom “Mind wandered during tasks” (DEP2) occupied the most central node in the network, while “Negative future optimism” (DEP5) held the position of the least central symptom. The strongest edge within the depressive symptoms network was found between “Negative future optimism” (DEP5) and “Unhappiness” (DEP8). Supplementary Figure S3 details the standardized estimates for node strength, betweenness, closeness, and expected influence, and Supplementary Figure S4 provides the bootstrapped confidence intervals (CIs) for the edge weights.

### 3.3 Combined internalized HIV stigma and depressive symptoms network model

**Figure 3:**
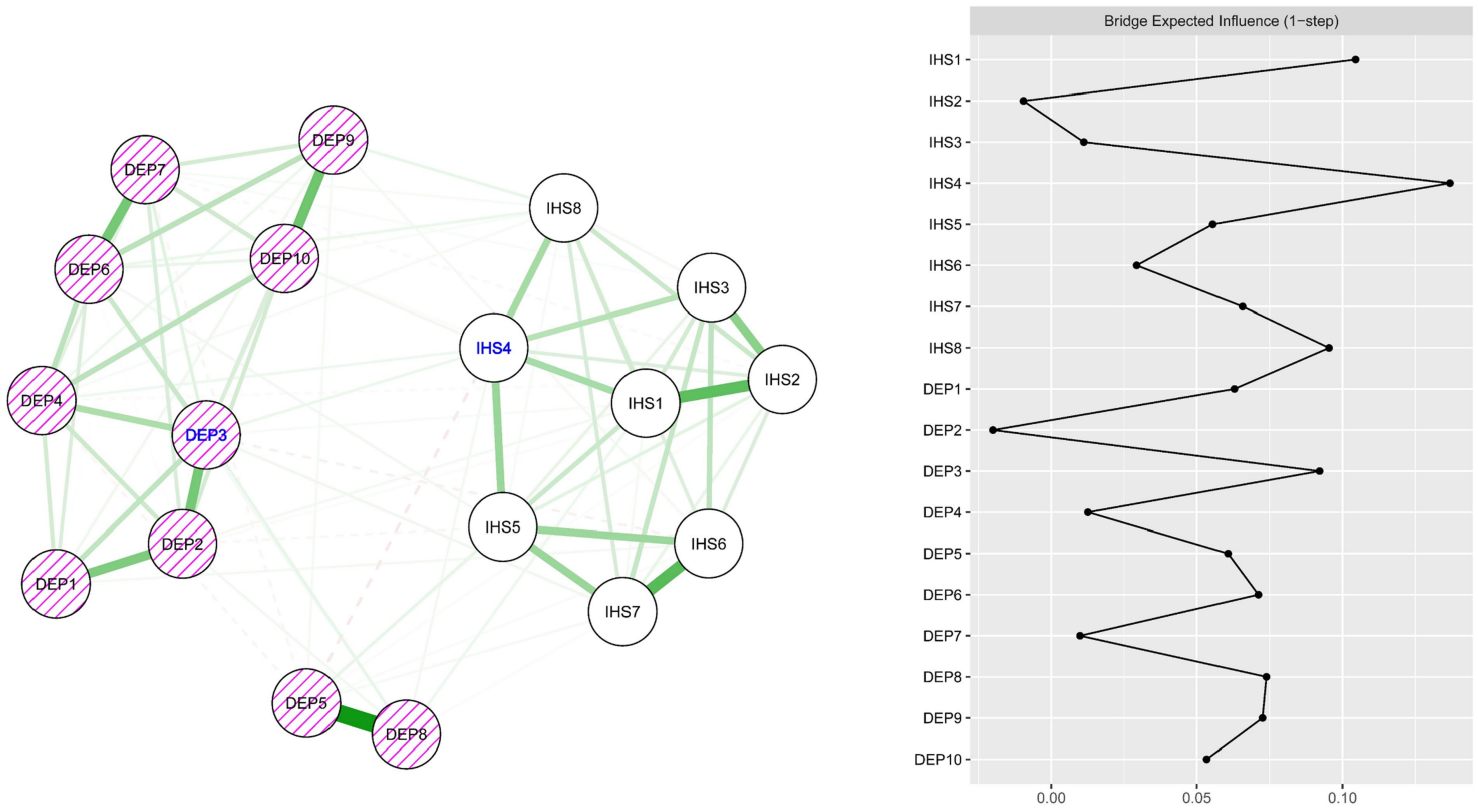
Combined regularized partial correlation network model for internalized HIV stigma (IHS) and depressive symptoms (DEP3) and bridge expected influence (BEI) values for each node. Bridge nodes with large BEI values are represented by blue text (IHS4 and DEP3).

Figure 3 presents the combined network model that connects internalized HIV stigma and depressive symptoms. The stability of the model is confirmed by CS-coefficient values for edge weights [CS (cor = 0.7) = 0.75] and for BEI values [CS (cor = 0.7) = 0.48]. Within this combined network model, the nodes “Inferiority due to HIV” (IHS4) from the internalized HIV stigma community and “Gloomy feelings” (DEP3) from the depressive symptoms cluster were highlighted as bridge nodes owing to their highest one-step BEI values. The metrics of node strength, betweenness, closeness, and expected influence are standardized and represented in Supplementary Figure S5. Furthermore, Supplementary Figure S6 illustrates the bootstrapped confidence intervals (CIs) corresponding to the edge weights.

## 4 Discussion

In this study, network analysis was utilized to explore the network structures of internalized HIV stigma, depressive symptoms, and their interactions in a large sample of 1,197 Chinese PLWH during the COVID-19 pandemic. This investigation is innovative because, to our understanding, it is the first to explore network models of internalized HIV stigma and depressive symptoms. It also examines the possible co-occurrence of internalized HIV stigma and depressive symptoms and identifies the bridge between these two psychological phenomena among the marginalized and vulnerable population.

Our study provides valuable insights into the network structure of internalized HIV stigma and depressive symptoms among PLWH during the COVID-19 pandemic. One of the key findings was that “ashamed of having HIV” emerged as the most central node within the internalized HIV stigma network, indicating its pivotal role in the interconnected system of stigma-related experiences. This suggests that shame associated with HIV diagnosis may significantly influence other stigma-related experiences and contribute to the overall burden of internalized stigma. Such a finding is consistent with previous research highlighting the detrimental effects of shame on individuals’ self-esteem and psychological well-being (41–44). The central role of shame in the internalized HIV stigma network underscores the need for interventions that specifically target and mitigate feelings of shame associated with an HIV diagnosis, especially when PLWH are facing public health crises. Our research recognizes that some components of internalized HIV stigma, such as the centrality of “ashamed of having HIV,” are not limited to the COVID-19 outbreak. Nonetheless, our investigation specifically sheds light on how the exceptional difficulties presented by the pandemic could have intensified and influenced these persistent factors of stigma and depression in individuals living with HIV. The COVID-19 pandemic has led to several challenges for PLWH, including amplified feelings of shame, guilt, and depressive symptoms due to increased social isolation, heightened health anxieties, and disruptions in healthcare services. Our network analysis, while based on established measures, contextualizes these phenomena within the specific challenges faced by PLWH during COVID-19, underscoring the pandemic’s impact on their mental health.

The centrality of “mind wandered during tasks” in the depressive symptoms network highlights the impact of cognitive dysfunction on PLWH’s daily functioning and mental health status. This finding aligns with previous research showing that cognitive difficulties are a common symptom of depression that can exacerbate other depressive symptoms and impede overall recovery (45–48). Cognitive difficulties can interfere with an individual’s ability to effectively engage in self-care behaviors, interpersonal interactions, and other activities critical to managing HIV and maintaining good mental health (49–51). Additionally, the process of mind wandering is often associated with a lack of focus, decreased productivity, and an overall diminished ability to engage in the present moment, all of which are common characteristics of depression (52–54). Frequent mind wandering may indicate a struggle to manage or control thoughts, which can lead to an increased experience of depressive symptoms (55, 56). Within the context of the COVID-19 pandemic, this finding suggests that pandemic-induced stressors, such as prolonged isolation and health anxiety, significantly worsen cognitive disruptions. Specifically, these factors have intensified PLWH’s difficulty in maintaining focus on daily tasks, a key aspect of depressive symptomatology.

Our network analysis also identified two critical bridge connections between the internalized HIV stigma and depressive symptoms communities—“inferiority due to HIV” and “gloomy feelings.” These bridge nodes suggest a reciprocal influence between these two psychological constructs, where feelings of inferiority due to HIV may lead to or exacerbate gloomy feelings, and vice versa. The bridge node “inferiority due to HIV” suggests that feelings of inferiority due to HIV status may act as a link between stigma and depressive symptoms. It may be that individuals who internalize societal stigma about HIV begin to perceive themselves as less than others because of their HIV status (57, 58). This sense of inferiority could subsequently trigger or exacerbate depressive symptoms. Inferiority could potentially lead to negative self-esteem and lack of self-worth, key aspects often associated with depressive states (59–64). On the other hand, “gloomy feelings” serving as a bridge node indicates that these symptoms are not only a consequence of internalized HIV stigma, but could also potentially feed back into the internalized HIV stigma cycle. Depression can lead to a negative cognitive bias, in which individuals interpret their experiences more negatively (65–67). Therefore, those with depressive symptoms may perceive their HIV status in a more negative light, thereby increasing the internalization of HIV stigma.

These bridge nodes highlight the possible bidirectional and complex relationship between internalized HIV stigma and depressive symptoms, suggesting a potentially cyclical and self-perpetuating system. The more an individual feels inferior because of their HIV status, the more susceptible they may become to depressive symptoms. In turn, these depressive symptoms could further increase feelings of stigma, creating a reinforcing loop. Recognizing the role of “inferiority due to HIV” and “gloomy feelings” as bridge nodes illuminates pathways through which HIV stigma and depressive symptoms may interact and influence each other. This insight underlines the importance of addressing both internalized HIV stigma and depressive symptoms in a holistic and integrated manner in mental health interventions, particularly in the context of the COVID-19 pandemic, which has exacerbated both of these challenges for PLWH.

### 4.1 In the context of COVID-19

Our study utilized network analysis to investigate the patterns of internalized HIV stigma and depressive symptoms among PLWH during the COVID-19 pandemic. The results revealed that “ashamed of having HIV” and “mind wandered during tasks” played central roles in the networks of internalized stigma and depressive symptoms, respectively. These findings underscore the significant influence of shame and cognitive dysfunction on the experiences of individuals living with HIV during the pandemic. Notably, the findings suggest that “inferiority due to HIV” and “gloomy feelings” play a critical role as bridge nodes, reflecting a potential bidirectional influence between internalized HIV stigma and depressive symptoms. The results suggest that the COVID-19 pandemic may have intensified these experiences, exacerbating the sense of inferiority and gloominess among people living with HIV. These findings highlight the intricate relationship between internalized HIV stigma and depressive symptoms during the pandemic, emphasizing the necessity for comprehensive and integrated mental health interventions. It is critical to address both internalized stigma and depressive symptoms, particularly given the supplementary stressors caused by COVID-19, including heightened social isolation and health anxieties.

### 4.2 Limitations and implications

Several limitations of this study must be acknowledged. First, the cross-sectional design of the study limits our ability to make causal inferences regarding the temporal characteristics and directional effects of associations between internalized HIV stigma items and depressive symptoms (18, 19). While our findings provide a preliminary empirical basis for future hypotheses, further longitudinal and experimental studies are needed to explore the temporal sequences and potential causal relationships between these psychological phenomena (20, 21). Second, the study relied on self-reported data, which may be susceptible to response bias. Future research would benefit from using multiple data collection methods, such as observer ratings, structured diagnostic interviews, and mixed methods, to minimize potential bias. Third, it should be noted that in the current study, we used the CESD-10 to assess participants’ depressive symptoms. Measurements with diagnostic function are recommended in future research exploring network analysis regarding depression in PLWH. Finally, the interpretation of which bridge symptoms are significant may be influenced by subjectivity, as bridge centrality does not necessarily indicate effect size (68). Therefore, additional research is needed to clarify the role and interpretation of bridge centrality in the context of internalized HIV stigma and depressive symptoms.

Despite the limitations, our research has significant implications for future strategies in public health and medical practice. In particular, this study highlights “inferiority due to HIV” and “gloomy feelings” as key bridging elements linking internalized HIV stigma and depressive symptoms among Chinese PLWH during the COVID-19 crisis. Expanding on these findings, understanding the central role of these bridge elements may provide a new focus for health professionals in addressing mental health concerns among PLWH. Therapeutic interventions should aim to address feelings of inferiority associated with HIV and manage depressive symptoms, such as cognitive behavioral therapy-based and mindfulness-based interventions (69–72). This approach can potentially break the cycle of stigma and depressive emotions, thereby improving mental health outcomes for this population (71–73). In a broader public health context, these findings highlight the need for stigma reduction initiatives. Programs aimed at improving societal attitudes toward HIV and providing mental health support to those affected could play an important role in reducing both internalized HIV stigma and related depressive symptoms (74). Increasing the availability and accessibility of mental health services for people living with HIV could help manage depressive symptoms more effectively (75). The health care community could use these findings to develop specific tools and resources to facilitate the management of feelings of inferiority and depressive symptoms among PLWH. This could include the development of psychosocial support groups, cognitive-behavioral interventions, or resilience-building programs tailored specifically for PLWH (74). Finally, our findings suggest that depression measurement in PLWH, particularly in the context of the COVID-19 pandemic, may benefit from the inclusion of items that capture the nuanced influence of HIV-related stigma on depressive symptoms and from a greater focus on symptom-specific measurement. These considerations could improve the accuracy and utility of depression measurement, thereby informing more effective interventions for this population. In addition, the implications of our study extend beyond the COVID-19 pandemic. The structures of internalized HIV stigma and depressive symptoms that we identified, along with their interactions, provide valuable insights for mental health interventions in any prolonged crisis or epidemic setting among PLWH. Understanding these intricate relationships aids in customizing strategies for alleviating depressive symptoms and internalized stigma, ultimately leading to improved mental health outcomes. This information is crucial for upcoming public health responses and mental health support structures, guaranteeing their suitability and efficiency during both pandemic and post-pandemic periods.

## Data availability statement

The raw data supporting the conclusions of this article will be made available by the authors, without undue reservation.

## Ethics statement

The studies involving humans were approved by the Institutional Review Board of University of South Carolina. The studies were conducted in accordance with the local legislation and institutional requirements. The participants provided their written informed consent to participate in this study.

## Author contributions

GY: Conceptualization, Data curation, Formal analysis, Methodology, Software, Validation, Visualization, Writing – original draft. SQ: Data curation, Investigation, Project administration, Resources, Supervision, Writing – original draft. XL: Data curation, Funding acquisition, Project administration, Resources, Supervision, Writing – original draft.
