# Supplementary material for: Bridging internalized HIV stigma and depressive symptoms among people living with HIV in China during the COVID-19 pandemic: a network analysis
Source: Front Public Health. 2024 Jan 5;11:1306414. doi: 10.3389/fpubh.2023.1306414 (PMC10796684; doi:10.3389/fpubh.2023.1306414)
Supplement: Supplementary file 1 [file Image_1.PDF]

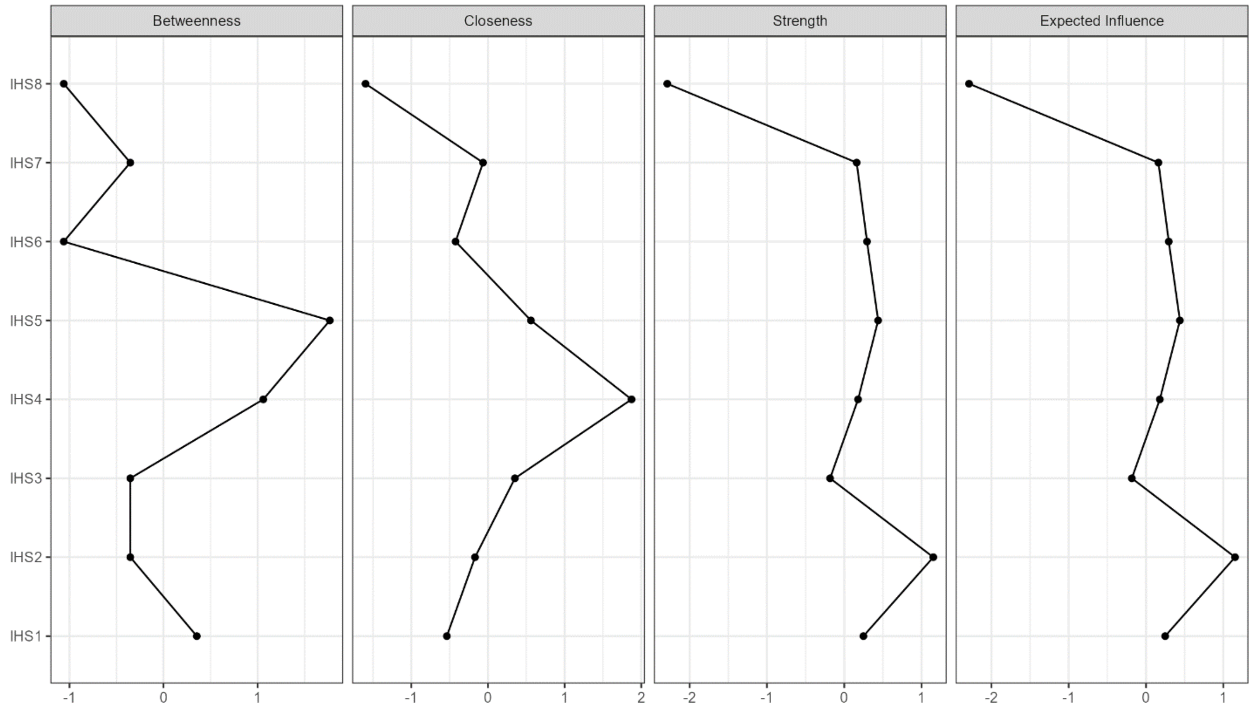

Fig. A.1. Node strength centrality, betweenness, closeness, and ExpectedInfluence estimates for internalized HIV stigma items. See Table 3 for item descriptions of the short codes

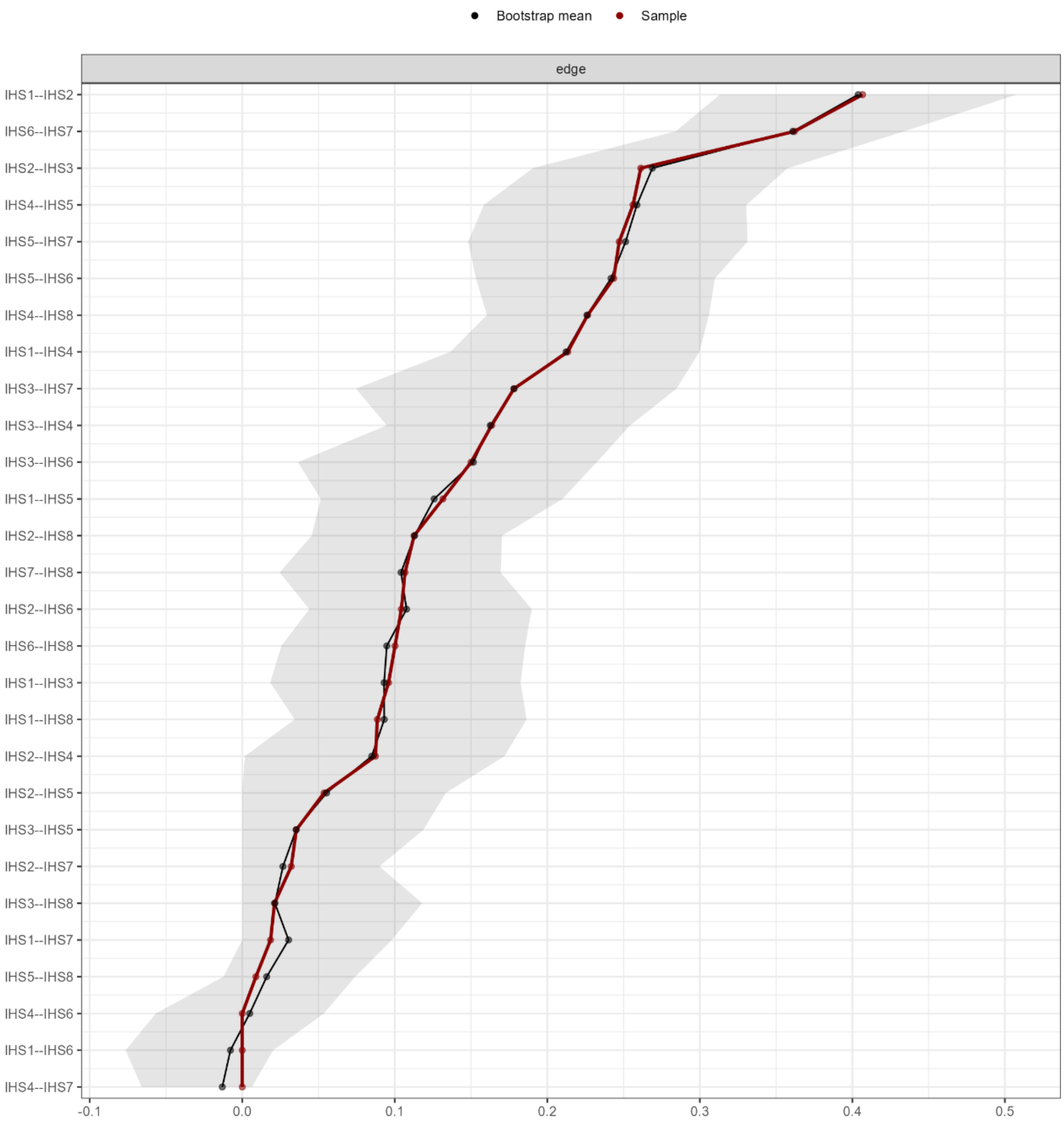

Fig. A.2. Bootstrapped confidence intervals (CIs) of the edge weights in the internalized HIV stigma network.

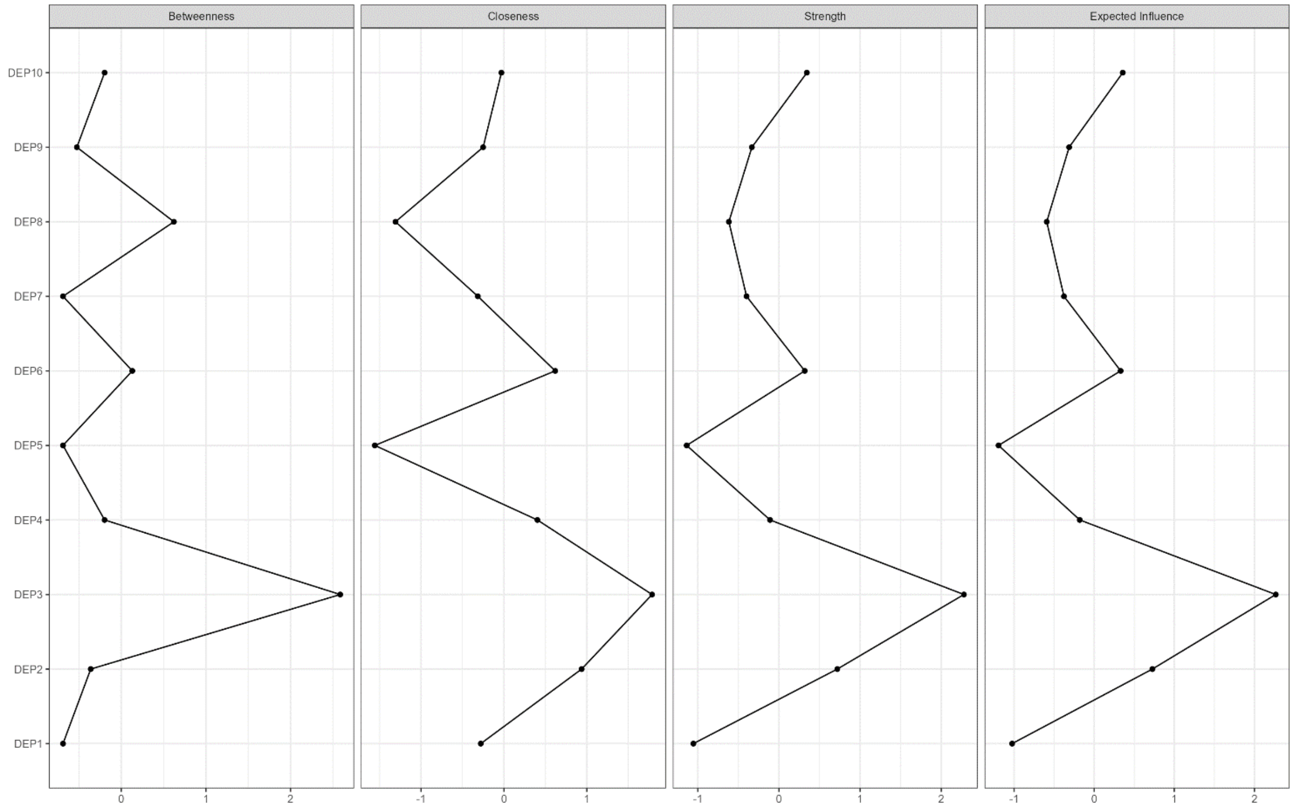

Fig. A.3. Node strength centrality, betweenness, closeness, and expectedinfluence estimates for depressive symptoms. See Table 3 for item descriptions of the short codes

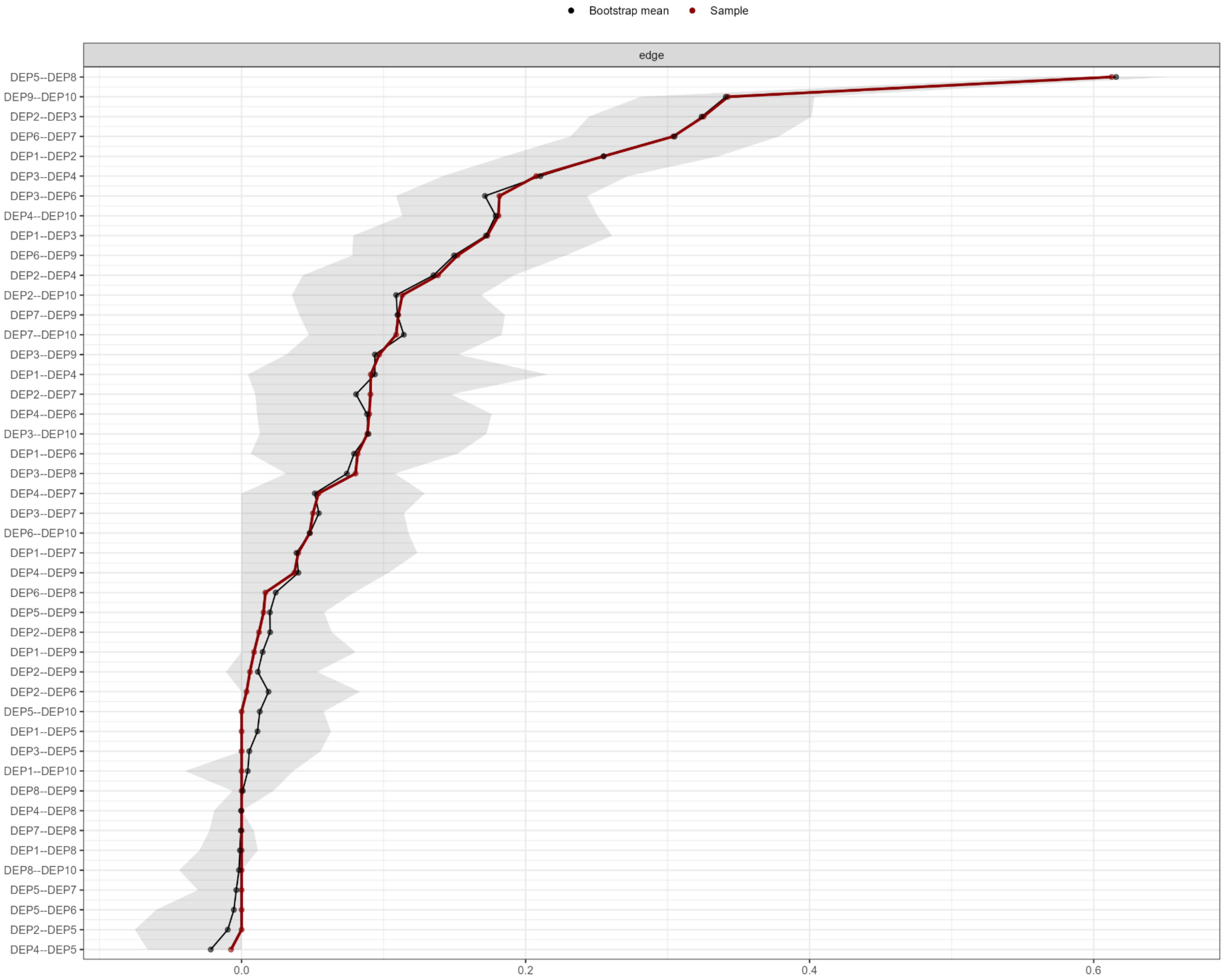

Fig. A.4. Bootstrapped confidence intervals (CIs) of the edge weights in the depressive symptoms network model.

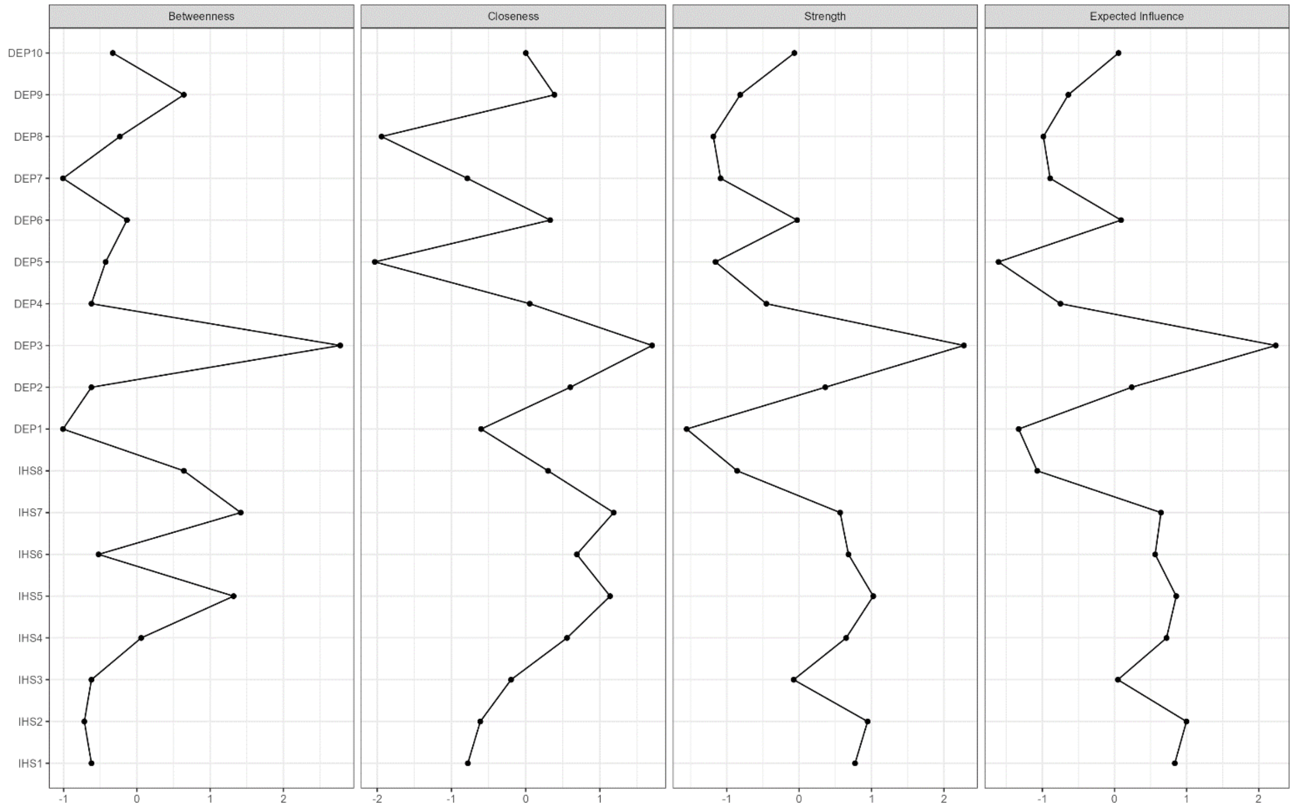

Fig. A.5. Node strength centrality, betweenness, closeness, and ExpectedInfluence estimates for the internalized HIV stigma and depressive symptoms items. See Table 3 for symptom descriptions of the short codes.

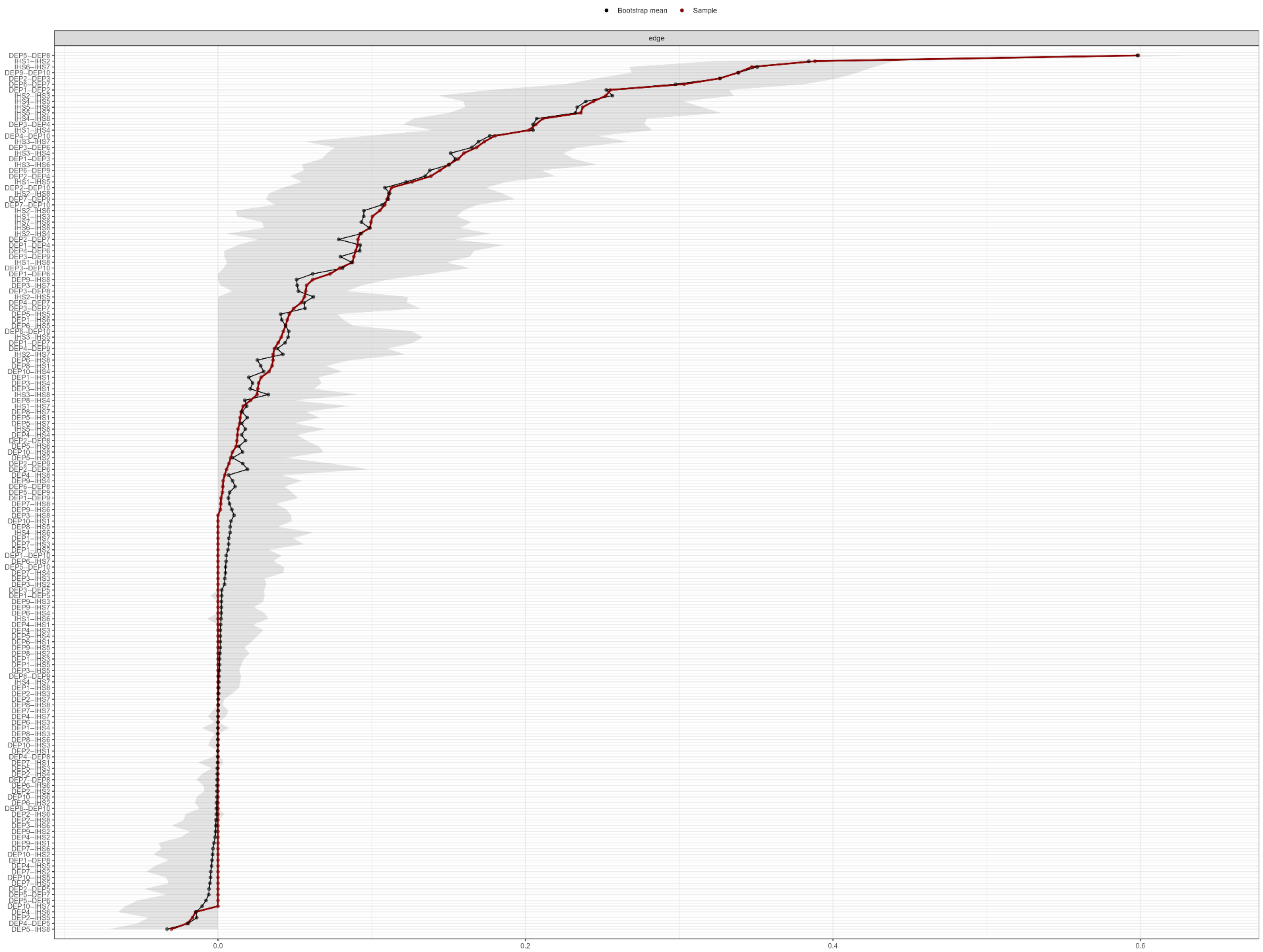

Fig. A.6. Bootstrapped confidence intervals (CIs) of the edge weights in the combined internalized HIV stigma and depressive symptoms model.

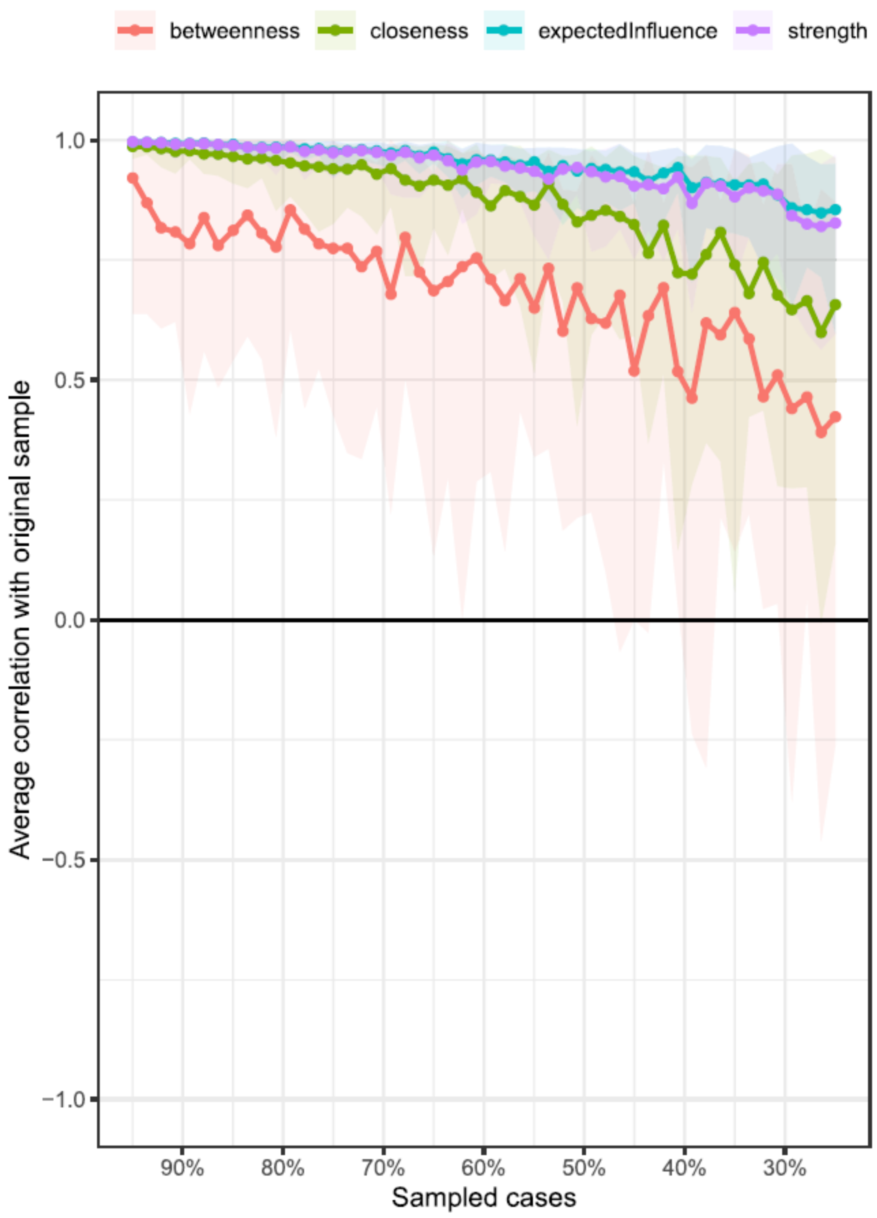

Fig. A.7. Bootstrapped node Betweenness, Closeness, Strength, and ExpectedInfluence of the estimated internalized HIV stigma network model.

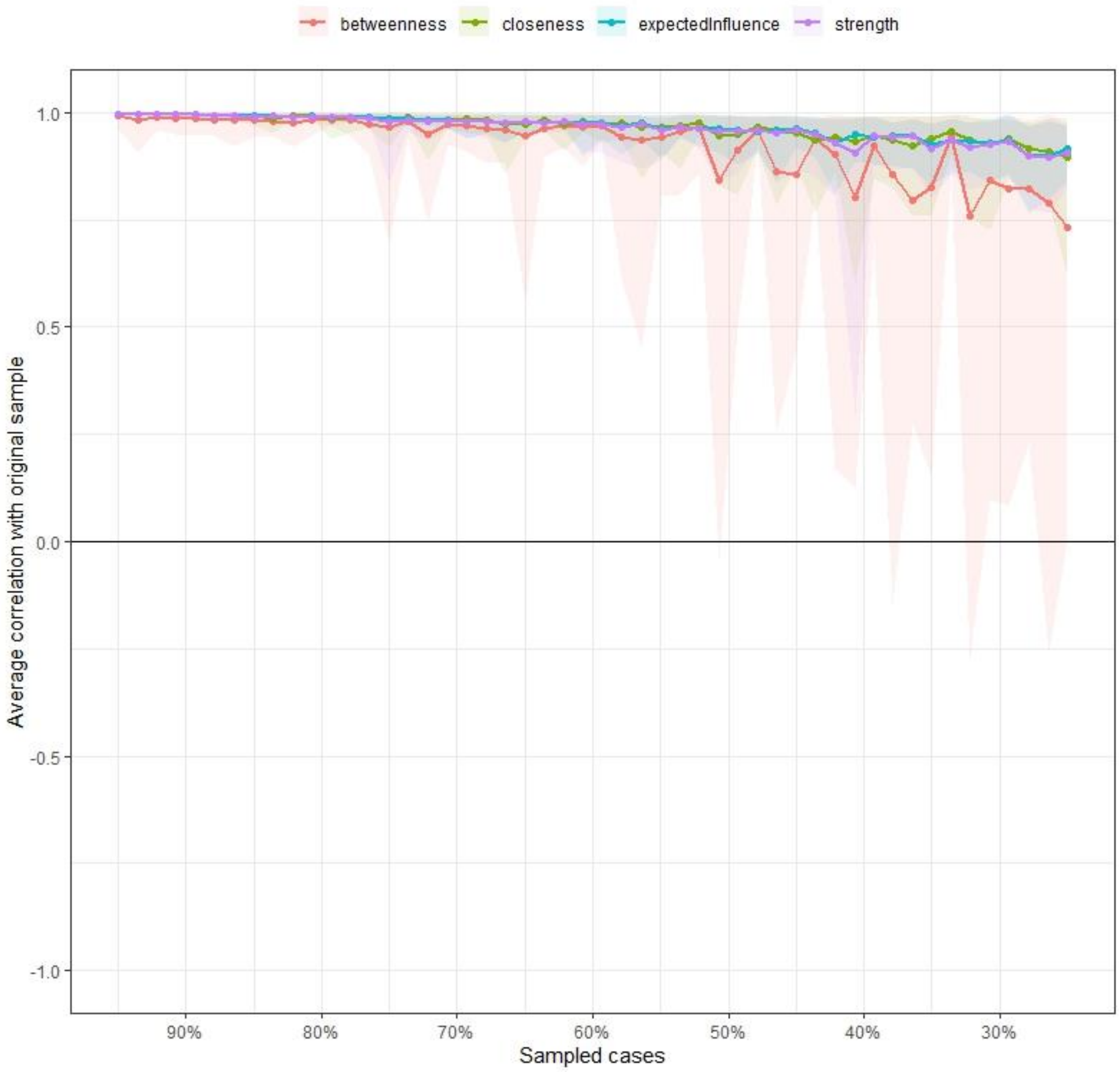

Fig. A.8. Bootstrapped node Betweenness, Closeness, Strength, and ExpectedInfluence of the depressive symptoms network model.

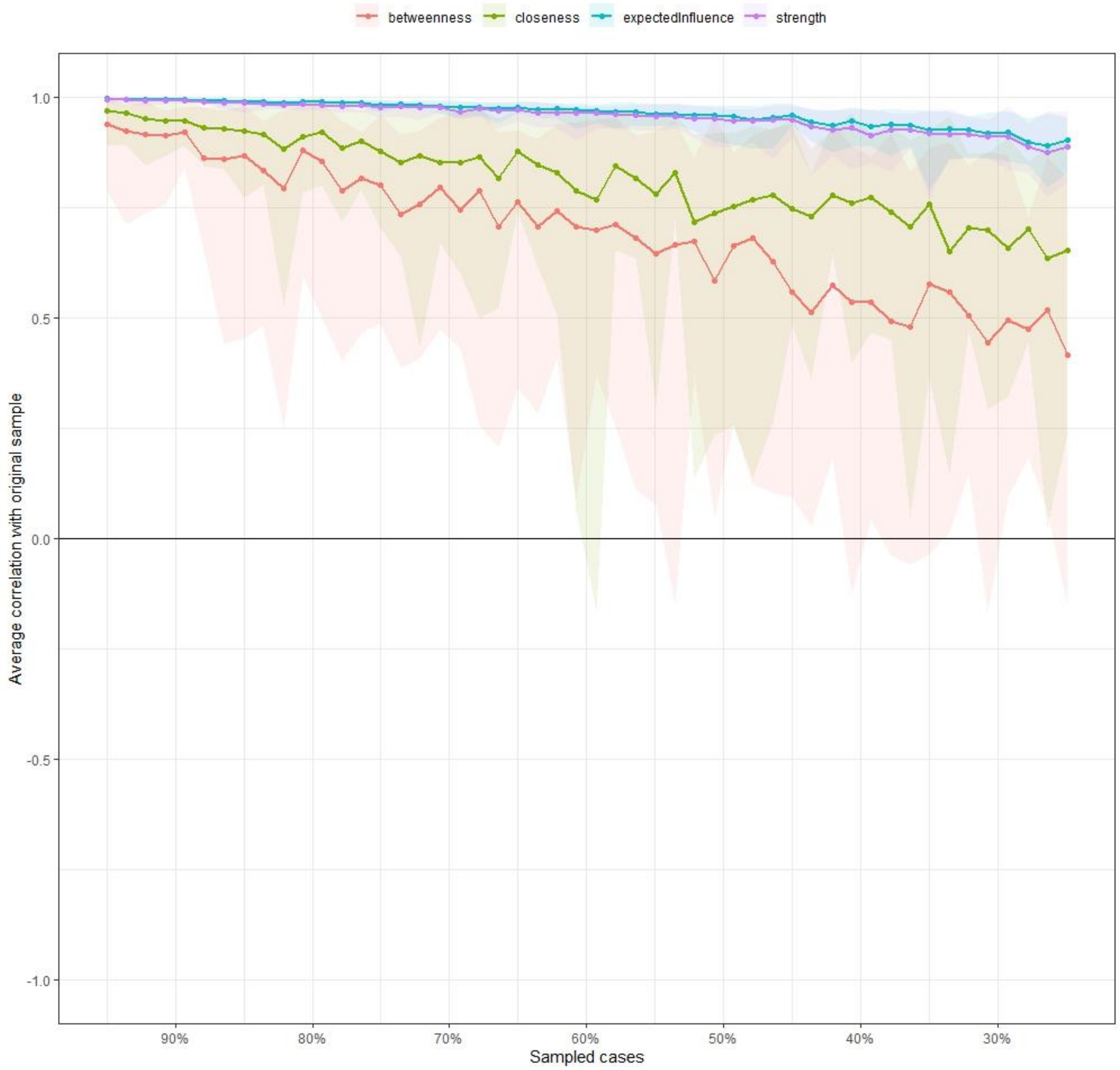

Fig. A.9. Bootstrapped node Betweenness, Closeness, Strength, and ExpectedInfluence of the estimated combined network model.
